# Disease burden of spinal muscular atrophy in Germany

**DOI:** 10.1186/s13023-016-0424-0

**Published:** 2016-05-04

**Authors:** Constanze Klug, Olivia Schreiber-Katz, Simone Thiele, Elisabeth Schorling, Janet Zowe, Peter Reilich, Maggie C. Walter, Klaus H. Nagels

**Affiliations:** Institute for Healthcare Management and Health Sciences, University of Bayreuth, Prieserstrasse 2, 95444 Bayreuth, Germany; Department of Neurology, Hannover Medical School, Carl-Neuberg-Strasse 1, 30625 Hannover, Germany; Department of Neurology, Friedrich-Baur-Institute, Ludwig-Maximilians-University of Munich, Ziemssenstrasse 1, 80336 Munich, Germany

**Keywords:** Spinal muscular atrophy (SMA), Health-related quality of life (HRQOL), Direct cost, Indirect cost, Informal care cost, Cost of illness (COI), Health care burden, Neuromuscular disease, Health services research

## Abstract

**Background:**

This study aimed at analyzing the economic burden and disease-specific health-related quality of life (HRQOL) of patients with spinal muscular atrophy (SMA) in Germany. SMA is a so far non-curable neuromuscular disease of the anterior nerve cells that causes high rates of morbidity and mortality.

**Methods:**

In a cross-sectional study we analyzed the cost of illness (COI) and factors that influence the direct, indirect and informal care costs of affected patients and their families by using standardized, self-developed questionnaires. We used the PedsQL™^©^ Measurement Model to analyze the disease-specific HRQOL of patients.

**Results:**

One hundred eighty nine patients with SMA types I to III aged <1 to 73 years were enrolled. The average annual COI was estimated at €70,566 per patient in 2013. The highest cost resulted in SMA I with significant lower costs for the milder phenotypes. Inversely, the self-estimated HRQOL increased from SMA I to SMA III. Major cost drivers were informal care cost and indirect cost incurred by patients and their caregivers.

**Conclusions:**

Although SMA requires high standards of care, there has been a distinct lack of health services research on SMA. Accordingly, our results significantly contribute to a more comprehensive insight into the current burden of SMA and quality of life status as related to SMA health services in Germany. In the light of innovative therapeutic interventions, our results suggest a notable potential for a reduction in overall COI and improvement of HRQOL if the therapeutic intervention leads to a less severe course of the disease.

## Background

Spinal muscular atrophy (SMA) is an autosomal recessive, inherited neuromuscular disease that affects the spinal anterior nerve cells. It leads to predominantly proximal muscle and diaphragm weakness and paralysis, along with respiratory distress, progressive disability, reduced working capacity and high health care needs. Infantile forms significantly reduce life expectancy [1, 2]. In >90 % of cases the disease is caused by homozygous deletions within the *survival motor neuron 1* (SMN1) gene on chromosome 5q13 [3]. SMA caused by SMN1 deletions is usually classified into three phenotypes characterized by age of onset and the best motor function achieved. SMA type I (Werdnig-Hoffmann) is the most common and severe subtype, and SMA type III (Kugelberg-Welander) with onset >18 months and achievement of independent walking represents the mildest one [4]. SMA is so far not curable; and patients need long-term symptomatic multidisciplinary medical care to maintain mobility, independency, ventilation and nutrition [5]. Living with SMA is challenging not only for patients but also for their families and caregivers, as well as medical personnel and the society. Economic analyses and health services research for SMA have not been conducted in any great detail to date, but are urgently needed with regard to emerging innovative therapies. To be able to compare health care expenditures for the current symptomatic treatment and care with costs of future curative therapies, cost evaluations are mandatory. Our study provides a detailed analysis of the cost of illness (COI) of SMA, differentiating between the SMA subtypes in Germany. However, the burden of SMA cannot be reduced to an analysis of cost items only. Considering the disease-specific challenges and the individual burden it is also necessary to analyze important aspects for the affected individual as health-related quality of life (HRQOL).

## Methods

### Participants and procedures

We conducted a Germany-wide cross-sectional study over a four months period from April to August 2013. Disease- and subtype-specific standardized questionnaires were self-developed in focus groups with input from clinicians, health-economists and patient representatives. The questionnaire comprised 109–123 items (depending on SMA type and respondent – patient or caregiver) including socio-demographic data, current/past occupation, health status and use of medical and care resources. For assessment of COI, respondents were asked to recall the consumption of resources over an exact time-period (e.g. drug treatment in the last week, hospitalizations within the last three months, constructional modifications since disease onset). The questionnaire had been utilized and pretested (*n* = 10) in a previous study assessing the health care burden of Duchenne muscular dystrophy (DMD) [6]. For assessment of the disease burden, we also analyzed the disease-specific HRQOL of patients either self- or proxy-reported by the Pediatric Quality of Life Inventory™^©^ (PedsQL™^©^), module for neuromuscular disorders, German version 3.0. This questionnaire was developed by Dr. James W. Varni [7, 8] and was translated into German by the Mapi Research Trust. The PedsQL™^©^ includes three major disease-related sections (‘problems with the neuromuscular disease’, ‘communication’ and ‘family resources’). The score had been validated for children and young adults (13–18) with SMA before [9–11]. However, we also utilized it in our adult patients, thereby ensuring best possible comparability of results. Patients with a genetically confirmed SMA (*n* = 265) were identified via the German SMA patient registry (www.sma-register.de), which was established within the network of excellence TREAT-NMD (www.treat-nmd.eu) in 2008 [12]. The paper-based questionnaire was distributed by post. For patients <16 years of age and/or highly care dependent patients, their caring relative or parent answered the questionnaire.

### Standard protocol approvals, registrations and patient consents

Patients and legal guardians/parents, respectively, gave their written consent to participate in this study, including the option of data withdrawal. The ethics board of the Ludwig-Maximilians-University of Munich approved the study.

### Data analysis

We retrospectively evaluated the total COI, comprising direct, indirect and informal care costs of individual patients and their families. Direct costs comprise the monetarily assessed disease-specific consumption of medical and non-medical resources (COR) [13]. Non-medical disease-specific costs include disease-related travel expenses, costs for legal advice and constructional modifications for example. Costs of hospitalization, drug treatment, rehabilitation services (e.g. physiotherapy, occupational therapy), medical aids and ventilation were aggregated as direct medical COI. The COR was assessed monetarily using empirical standard cost for Germany [14, 15], official price lists [16] and patient reported data such as cost for constructional modifications, legal advice or cost for housing. By anticipating a stable demand for health care resources, we estimated mean direct cost per year.

Informal care costs represent time spent for care by relatives, whereas indirect costs are productivity losses to society due to absenteeism, invalidity or premature death [13]. As these cost components are closely related, we analyzed informal care cost for non-working parents only to prevent double accounting of loss of productivity and care times of working parents. To analyze the indirect COI, we assessed the employment status of patients and parents/caregivers and evaluated disease-related productivity losses of patients and/or parents by considering absenteeism, invalidity or changes in their working situation.

Cost was estimated in Euros for 2013. Statistical analyzes were conducted using SPSS® test procedures (Mann–Whitney-U; *T* test). We only included complete and nearly complete questionnaires; any missing values were marked and excluded from the particular analysis.

## Results

### Demographics

One hundred eighty nine out of 265 invited SMA patients/parents were enrolled in this study (response rate 71 %). Patients had a median age of 19 years (range 0–73; Table 1); 59 % of the patients were male. All patients with SMA I and the vast majority of those with SMA II (92 %) and III (89 %) were covered by a statutory health insurance, in line with the typical distribution within the German population [17]. Differences between SMA types were seen in patients’ characteristics such as family status, education level, employment status and care level^1^ (Table 2). The majority of patients quit work or reduced working time when their best self-reported motor performance was reduced to sitting (SMA II: 100 %; SMA III: 39 %) or when walking became impaired (SMA III: 61 %, data not shown). Furthermore, parents of more severely affected children had to quit their jobs or reduce working times more frequently, which resulted in lower gross salaries per year (Table 2).

**Table 1:** Patient demographics: Response rate, age and SMA-related characteristics

	SMA subtype			
	Total	I	II	III
Response rate				
Total patient number	265	20	115	130
Enrolled respondents	189	12	73	104
Response rate [%]	71	60	63	80
Ratio of patients/parents among respondents [%]	52/48	0/100	30/70	74/26
Age of enrolled patients				
Min. [years]	0	0	2	2
Max. [years]	73	7	56	73
Median [years]	19	1	11	33
Current motor function				
N	177	9	69	99
Walk [%]	33	0	0	59
Sit [%]	47	11	65	38
None [%]	20	89	35	3
Wheelchair use				
N	154	4	51	99
Always [%]	61	50	100	41
Sometimes [%]	14	0	0	21
None [%]	25	50	0	37
Feed tube use				
N	177	9	69	99
Yes [%]	6	22	10	1
No [%]	94	78	90	99
Spine surgery				
N	176	9	68	99
Yes [%]	23	0	22	8
No [%]	153	100	78	92
Non-invasive ventilation				
N	174	9	69	96
Always [%]	1	0	1	0
Sometimes [%]	12	22	22	4
None [%]	87	78	77	96
Invasive ventilation				
N	174	8	69	97
Always [%]	1	13	0	0
Sometimes [%]	0	0	0	0
None [%]	99	88	100	100

Differentiation by SMA subtypes (I = Werdnig-Hoffmann, II = intermediate, III = Kugelberg-Welander). This differentiation into subtypes is used for all following tables and figures. SMA-related characteristics of enrolled patients (motor function, spine surgery, use of wheelchair, feed tube use and ventilation) originate from the German SMA registry at the time of execution. Because of rounding, percentages might not add up to exactly 100 %. N = number of patients

**Table 2:** Patient demographics: Family status, education, employment status and patient care levels

	Patients			Parents		
	I	II	III	I	II	III
Family status [%]						
Widowed	-	0	1	-	2	-
Divorced	-	0	11	-	6	11
Married	-	5	33	75	75	78
In a partnership	-	18	9	17	16	11
Unmarried	-	77	46	8	2	-
Education [%]						
No qualification	-	-	3	-	2	4
School	-	73	63	75	62	70
University	-	27	35	25	36	26
Completed professional training	-	60	79	92	92	85
Employment status [%]						
Non-working	-	52	26	-	6	7
Currently working						
Self-employed	-	-	4	18	4	-
Employed	-	38	49	27	60	74
Reduced working time	-	13	21	100	58	10
Quit working life	-	10	21	55	29	19
Care levels [%]^a^						
No care level	20	1	37	-	-	-
Care level 1	10	12	20	-	-	-
Care level 2	20	27	31	-	-	-
Care level 3	50	59	13	-	-	-
Gross salary per year [€, mean (SD)]	-	28,496 (22,218)	38,437 (23,055)	22,813 (14,182)	28,359 (21,015)	35,347 (27,417)

These demographic parameters served as a basis for the indirect cost calculation of patients and their parents. The classification into care levels (level 1–3) represents the individual classification within the German health care system; a higher number indicates greater needs (^a^care level 1: at least 90 min/day of which at least 45 min account for basic care needs (personal hygiene, feeding, mobility); care level 2: at least 3 h/day of which at least 2 h account for basic care needs; care level 3: at least 5 h/day of which at least 4 h account for basic care needs [36]). Patients/parents collated in the category ‘quit working life’ quit their working lives due to the disease. Those who are ‘non-working’ additionally represent pensioners, trainees and students. Data is presented as % or mean (SD). Lines indicate not applicable answers. Because of rounding, percentages might not add up to exactly 100 %

### Direct COI

**Fig. 1:**
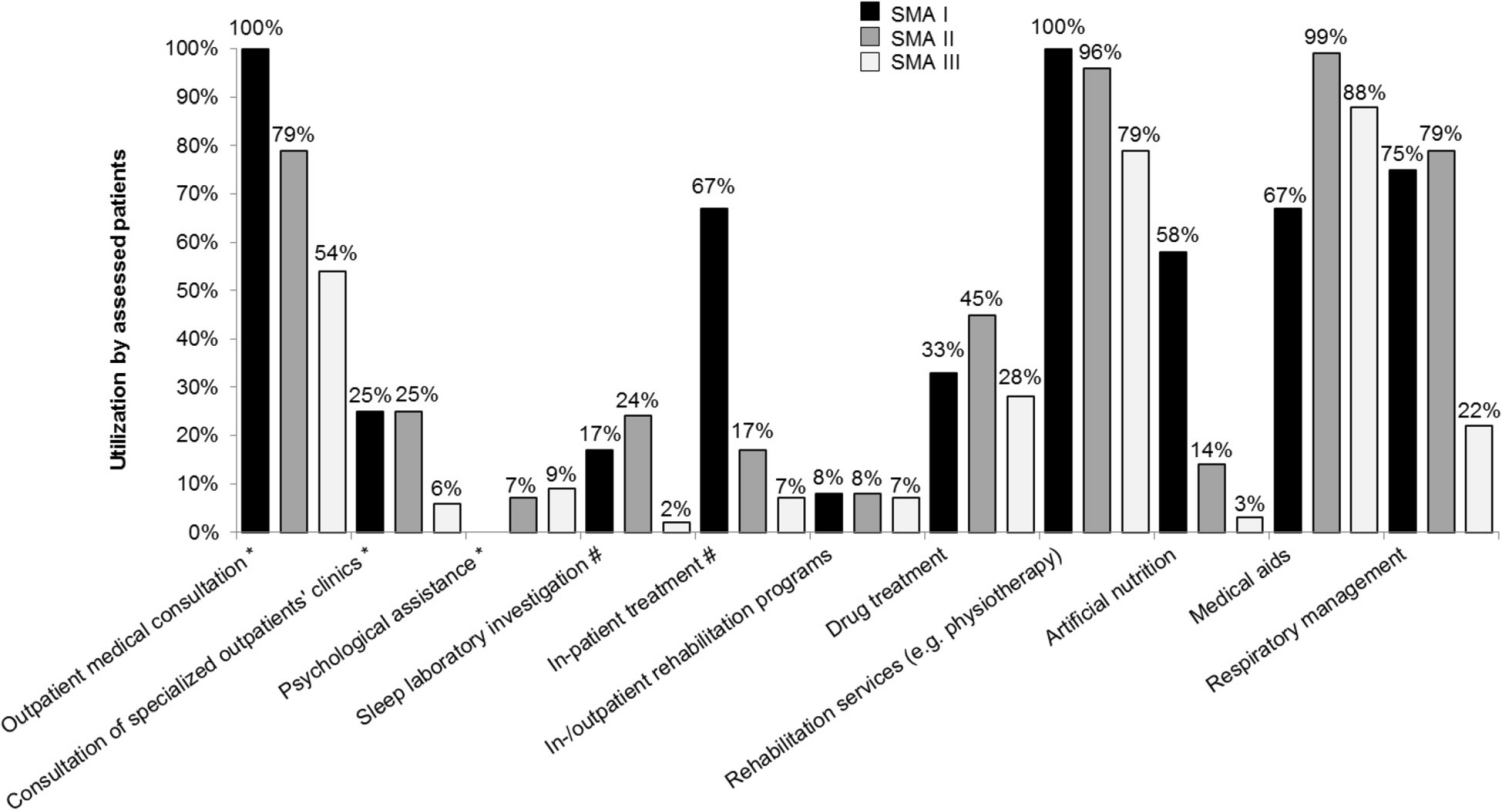
Consumption of resources (COR) of direct medical services of SMA patients. The COR of direct medical services is differentiated by SMA subtypes and shown in % of total patients. ^*^Collated as ‘outpatient medical cost’. ^#^Collated as ‘inpatient medical cost’

The direct medical COI was analyzed based on the COR of every individual patient. SMA I patients displayed the highest COR, mainly in form of outpatient medical consultations, in-patient treatment and artificial nutrition (Fig. 1). Less severely affected patients showed a decrease in the COR; however, utilization of sleep laboratory diagnostics, drug treatment, medical aids and respiratory management were most prominent in SMA II, and the highest need for psychological assistance was seen in SMA III.

Based on the COR, the total annual direct medical COI was estimated at a mean of €14,342 per patient (20 % of total COI) with significant differences between the SMA subtypes. The direct medical COI was more than three times higher in SMA I (€53,707/y per patient) than in SMA II and III (€15,305/y and €9,125/y per patient resp., *p* < 0.001). SMA I patients produced the highest cost in nearly all assessed categories (apart from medical aids and attendance in rehabilitation programs). Costs for inpatient medical treatments and for medical aids (8 % and 5 % respectively of total COI) were identified as the main cost drivers.

In contrast, direct non-medical COI (e.g. care cost, disease-related investments) was about 2.8 times higher than the mean direct medical COI (€40,378/y per patient, 58 % of total COI, Table 3). Direct non-medical cost was most prominent in SMA II with significant differences between SMA I/II and SMA III (*p* < 0.05). Informal care was the most important non-medical cost driver in all SMA subtypes (29 % of total COI). Total overall care effort was estimated at 7.8 h/day (of which parents provided 7 h/day) and turned out to be most relevant in SMA I (15 h/day; SMA II: 11 h/day; SMA III: 4 h/day). Mainly parents (69 %) and partners (21 %, not in SMA I) provided informal care. While informal care costs decreased from SMA I to III, total mean direct non-medical COI increased from SMA I to SMA II due to higher costs for housing, personal assistance and constructional modifications in SMA II.

**Table 3:** Cost of illness (COI) per patient for SMA [in €/y]

	Mean	Ratio of total COI [%]	SMA subtype		
			I	II	III
Outpatient medical costs	331 (579)	0 %	463 (455)	392 (496)	274 (640)
Inpatient medical costs	5,634 (24,193)	8 %	39,972 (81,057)	4,454 (11,892)	2,488 (10,730)
Rehabilitation costs (in-/outpatient)	822 (3,535)	1 %	594 (2,059)	971 (3,901)	745 (3,423)
Drug treatment costs	189 (527)	0 %	389 (1,232)	245 (563)	126 (334)
Costs for use of rehabilitation services	2,050 (2,094)	3 %	3,488 (4,734)	2,149 (1,641)	1,814 (1,849)
Costs for artificial nutrition	243 (1,157)	0 %	1,940 (3,351)	247 (1,139)	90 (573)
Costs for medical aids	3,451 (2,450)	5 %	1,648 (1,628)	4,385 (2,133)	3,003 (2,508)
Costs for respiratory management	1,678 (2,760)	2 %	5,698 (5,882)	2,548 (2,280)	594 (1,713)
**Total direct medical COI**	**14,342 (26,379)**	**20 %**	**53,707 (81,809)**	**15,305 (14,220)**	**9,125 (14,330)**
Costs for housing	12,854 (38,639)	18 %	10,160 (26,569)	20,001 (50,000)	8,173 (29,107)
Costs for personal assistance for school and work attendance	2,162 (6,694)	3 %	0	4,301 (9,513)	958 (3,794)
Travel expenses	2,800 (6,637)	4 %	2,068 (1,866)	2,040 (2,525)	3,424 (8,661)
Informal care costs	20,170 (28,924)	29 %	31,542 (22,541)	28,571 (35,107)	12,902 (22,173)
Costs for legal advice	9 (37)	0 %	0	12 (37)	8 (39)
Costs for constructional modifications to house	1,860 (3,408)	3 %	833 (2,887)	2,882 (4,511)	1,254 (2,133)
Costs for constructional modifications to automobile	1,116 (2,577)	2 %	1,455 (4,824)	1,751 (3,252)	601 (1,193)
Other expenditures	129 (782)	0 %	20 (69)	110 (847)	156 (783)
**Total direct non-medical COI**	**40,378 (47,464)**	**58 %**	**45,957 (39,044)**	**58,607 (54,214)**	**26,940 (38,381)**
**Total direct COI**	**54,721 (59,210)**	**78 %**	**99,664 (104,071)**	**73,911 (60,597)**	**36,064 (42,753)**
Indirect cost patients	20,275 (22,857)	29 %	0	17,016 (18,782)	20,906 (23,645)
Indirect cost parents	13,204 (17,264)	19 %	13,959 (11,582)	19,034 (19,891)	4,195 (9,637)
**Total indirect COI**	**15,845 (20,067)**	**22 %**	**8,143 (11,173)**	**16,356 (18,971)**	**16,376 (21,511)**
**Total COI**	**70,566 (62,725)**	**100 %**	**107,807 (102,082)**	**90,267 (65,467)**	**52,440 (48,000)**

Data presented as mean (SD) in 2013 in € per year. Because of rounding, percentages might not add up to exactly 100 %. Costs for housing include e.g. professional care, domestic aids; other expenditures comprise patient-reported data for expenditures such as for special clothes for wheelchair users, podologic care etc.

In total, overall mean direct COI added up to €54,721/y per patient with significant differences between the SMA subtype I (€99,664/y per patient) to III (€36,064/y per patient, *p* < 0.001) and SMA II (€73,911/y per patient) to SMA III (*p* < 0.001, Table 3).

### Indirect COI

The indirect COI reflects the economic loss of productivity of patients and/or their parents caused by absenteeism, invalidity or changes in their working situation. Overall mean annual indirect COI added up to €15,845 per patient (Table 3). The highest productivity loss was found in SMA III patients. Parents seemed to be more limited in their careers and their productivity in SMA I and II caused by the patients’ younger age at disease manifestation.

### Total economic burden

Taking all described cost factors into account, the overall mean total COI was estimated at €70,566/y per patient. The most relevant disease burden was found in SMA I (€107,807/y per patient) with a decrease towards SMA II (€90,267/y) and SMA III (€52,440/y, Table 3) and a significant difference for SMA I and II compared to SMA III (*p* < 0.05). Using recent prevalence data from Northern England [18], we approximated the total economic burden caused by SMA in Germany at €106.2 million with the highest prevalence-based societal burden in SMA II (€41.4 million/y, Table 4).

**Table 4:** Total economic burden in Germany

		SMA subtype		
	Total	I	II	III
Prevalence [per 100,000] [18]	1.87	0.10	0.57	0.64
Total German population [in 2013] [37]	80,511,300			
Approx. patient number in Germany [in 2013]	1,506	81	459	515
Total COI per patient [in €/y]	70,566	107,807	90,267	52,440
Total national cost [in €/y]	106,241,507	8,679,665	41,424,872	27,021,093

National economic burden is based on prevalence data applying to Germany in 2013. Costs are presented in € for the year 2013

### Disease-specific health-related quality of life (HRQOL)

**Fig. 2:**
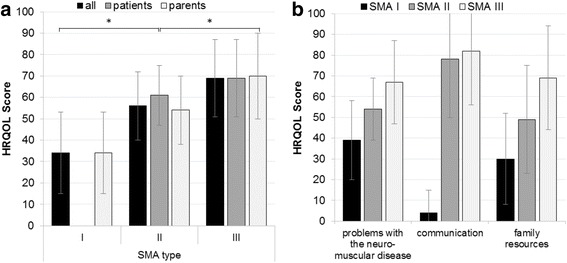
Disease-specific health-related quality of life (HRQOL) of SMA patients. Disease-specific HRQOL was assessed by PedsQL™^©^ Measurement Model, module for neuromuscular disorders, German version 3.0. Higher scores indicate better HRQOL. HRQOL was either patient self-reported or proxy-reported (**a**) and subdivided into the disease-specific sections: problems with the neuromuscular disease, communication and family resources (**b**). Mean scores are presented with standard deviations. Scale with 0 = min. and 100 = max. * indicates significant differences (*p* < 0.001)

Patients with SMA III assessed their disease-specific HRQOL as fairly high (self-reported), while SMA I patients had a low proxy-assessed HRQOL (69 vs. 34 on a scale with 0 = min. and 100 = max.; *p* < 0.001, Fig. 2a). We did not see any significant differences in the self- or proxy-evaluation of HRQOL in SMA II and III (*p* > 0.05). The most severe handicaps (along with lower scores) were identified in the qualitative test dimensions ‘problems with the neuromuscular disease’ and ‘problems with family resources’, while ‘communication’ was not so severely impaired (Fig. 2b). In all test dimensions, the scores increased from SMA I to III, indicating an increasing disease-specific HRQOL.

## Discussion

In this study, we performed a comprehensive analysis of the disease burden in SMA by evaluating direct and indirect cost as well as the disease-specific HRQOL. Our results revealed a mean direct COI of €54,721/y per patient which was about 14 times higher than the average health expenditure per patient in Germany in 2013 [19].

The highest overall demand for medical and care services was identified in SMA I, resulting in significantly higher cost compared to that of SMA II and III. Nevertheless, SMA I patients utilized respiratory devices and sleep laboratory tests to a lower extent than SMA II patients. Although respiratory failure is the most frequent cause of death in these young children [4], we found that not all of them were ventilated, which is in line with the patient characteristics within the German SMA registry (Table 1). The low ventilation rates in SMA I patients in Germany could be attributed to differences regarding the availability of expert SMA treatment centers, or different preferences of physicians and parents towards ventilation [12]. The use of medical aids seemed to be of secondary importance in SMA I, maybe due to the reduced physical development of SMA I patients. More importantly, this result might even suggest a possible shortage of medical aids (e.g. care aids or adapted beds) for this age group. The results of our study contribute to the transparency of the current consumption of resources of SMA patients in Germany. The use of psychological assistance turned out to be low in general, although previous publications showed a need not only for patients but also for the parents faced with a devastating disease affecting their kids [20]. Moreover, access to inpatient and outpatient rehabilitation programs seemed to be low, possibly revealing a supply gap in specialized rehabilitation centers.

As far as we know, this is the first study conducting a detailed health services evaluation to investigate the economic burden of SMA in Germany. Larkindale et al. analyzed the COI of different neuromuscular diseases (SMA, ALS, DMD, DM) in the US in 2010. However, COI results for SMA were not mentioned in the publication due to the small sample size and difficulties in an adequate classification of SMA subtypes [21]. This emphasizes the need for a detailed analysis of a genetically defined and comprehensively diagnosed group of SMA patients and highlights the importance of our study. Nevertheless, the results for SMA were published online by the US muscular dystrophy association (MDA). In contrast to our findings, higher costs were found in the patient group with diagnosis before/at age 3 ($184,647 per patient), but lower costs for patients with later onset ($45,750 per patient) [22]. These discrepancies may result from (1) differences in the origin of data (commercial, Medicare, patient-reported), (2) the definition of different subgroups (age at diagnosis vs. defined SMA subtypes), (3) the methodological differences in valuing COR, (4) general disparities between the US and German health care systems, e.g. incentives for both health care providers and patients or compensation systems, (5) differences in standards of care as recently analyzed by Bladen et al. [12] and (6) the varying characteristics of the populations with regard to morbidity and demography. These factors may influence country-specific COR and resulting cost and therefore limit the transferability and comparability of results between different countries [23]. That is precisely why our findings cannot easily be translated to other countries or other diseases. Nevertheless, the MDA study roughly confirms our results of higher cost in SMA I/SMA with early onset, which result from the higher level of COR and the care needed by these severely affected patients [22]. We previously reported similar results for dystrophinopathies in Germany and showed that health care expenditures increased with the progression of the disease and the increasing loss of an individual’s self-dependency [6].

According to our results, SMA type I patients caused the highest informal care cost, reflecting both the severity of the disease and greater need for care. SMA I patients typically have a drastically shortened life expectancy of <2 years [24, 25]. Even healthy babies and young children are demanding with regard to the attention and care that needs to be provided by the parents. Although we have assessed the informal care along with its cost in SMA, the comparability regarding the effort which is usually devoted to healthy children remains limited because of lack of publications in this field. Recently, COI analyses assessed informal care as a main cost driver in neuromuscular diseases, showing an increasing loss of family income together with increasing care need and dependency [6, 21, 26]. These findings fully apply to SMA. We found that a high proportion of working parents (esp. of SMA I patients) had to reduce or even quit their jobs to be able to care for their affected child, leading to a reduced family income. We estimated the average annual informal care cost per patient at €20,170. Since we excluded working parents from our care cost estimation to prevent double counting of indirect cost and informal care cost, our results may even underestimate real care expenditures (for example, the average annual informal care cost per patient including working and non-working parents is much higher at €34,871). Besides care cost, indirect cost was previously described as a major cost driver in dystrophinopathies [6, 26] and our results show similar findings in SMA. We estimated that indirect cost was most prominent in SMA II and III due to the loss of productivity of patients and/or their parents. Moreover, partners took over a major part of care in the group of adult patients, mainly in SMA III. Since we only analyzed the employment status of the patient itself or of one parent, indirect cost may be much higher when taking the second parent and/or other family members into account.

Altogether, we estimated a total economic burden of €106.5 million per year in Germany using prevalence data from Northern England [18]. Obviously, more precise assessments of country-specific epidemiologic data are urgently needed. One major step might be the implementation of patient registries in rare diseases like SMA. For this study, we utilized the German SMA patient registry (www.sma-register.de) [12] resulting in a response rate of >70 %, which is a very good result for a cross-sectional study dealing with sensitive areas of life such as cost, handicaps and individual problems.

Patient-reported outcomes (PRO) as HRQOL help to understand the perceived health state from a patient perspective, e.g. the individual impairment resulting from symptoms and disabilities, and reveals impacts on other dimensions of independent living [27]. Particularly in chronic diseases, HRQOL results can illustrate needs and shortcomings in health care, hopefully leading to improvements in services for adult [28] and pediatric/adolescent patients [29, 30]. In our study, HRQOL increased from SMA I to the milder SMA III phenotype, which is consistent with the results of a previous study in SMA II and III, in which a higher HRQOL in SMA III compared to SMA II had been shown [31]. Interestingly, in a Brazilian study, the self-reported HRQOL of children with SMA II/III (aged >4y) was irrespective of motor ability and SMA subtype (SMA II: 55.85 vs. SMA III: 52.94) [32]. However, they utilized the Autoquestionnaire Qualité de Vie Enfant Imagé (AUQEI) to assess HRQOL, a generic tool covering function, family, leisure, autonomy and other parameters, while we used the disease-specific instrument PedsQL™^©^to better determine differences between the SMA subtypes. Additionally, the assessment of different disease-related dimensions allows for a more precise comprehension of the important problems of patients in their daily lives. Thus, SMA had the most important impact on the dimensions ‘problems with the neuromuscular disease’, which encompasses disease-associated handicaps, and, secondly, ‘family resources’, which is related to familial financial and social aspects. The results of our study are confirmed by the results of a recent investigation in the Czech Republic in which the disease-specific HRQOL in SMA patients aged 3–18 years was similarly analyzed with the PedsQL™^©^ 3.0 Neuromuscular Module [33].

A potential limitation of our study might be a bias due to the utilization of a SMA patient registry to recruit study participants. Patients and families join the registry voluntarily; therefore, highly compliant and dedicated participants may be overrepresented. Furthermore, although SMA I is the most common SMA subtype, the life expectancy of patients is very limited. Given the low prevalence and small number of cases in this subgroup, our cost data for SMA I must be seen against this background. Additionally, in a retrospective study, recall bias may be a systematic error when estimating COI based on patient reported data from the past. Moreover, we used minimum prices to estimate the economic burden, possibly leading to the underestimation of the exact COI, and as mentioned above, the indirect cost may be much higher particularly when taking the impact on more than one family member into account.

In summary, our study provides the first comprehensive economic analysis of SMA in Germany from the perspective of patients, their families and society. Different innovative therapies are currently being investigated ‘from bench to bedside’, e.g. gene therapy, molecular therapy with antisense oligonucleotides, and small molecules, which hopefully will soon be available to treat this devastating disease [34, 35]. In this context, our study results show that new innovative therapies modifying the severity of SMA into a milder phenotype have the potential to reduce COR together with COI. However, innovative therapies may go hand in hand with high cost due to research and particularly clinical development activities. Considering this, a precise estimation of overall cost is not feasible. Modifying the severity of SMA towards a milder phenotype may be connected with improvements in patients’ quality of life. Although COI in SMA II was seen to be almost as high as in SMA I, SMA II patients showed a significantly higher quality of life. This further underlines the huge need for early-stage treatment and adequate support to reduce COR and to improve HRQOL.

## Conclusions

We analyzed the disease burden of SMA in Germany from a health economic perspective. This study is highly important for patients, families and caregivers as well as for the pharmaceutical industry. Concerning emerging therapies, giving equal priority to assessing the clinical and health economic situation may facilitate the translation of clinical research results into clinical practice. Importantly, our study contributes to a more comprehensive understanding of health care delivery issues related to SMA and neuromuscular diseases in general.

## Abbreviations

ALS: amyotrophic lateral sclerosis
AUQEI: autoquestionnaire qualité de vie enfant imagé
BMD: becker muscular dystrophy
COI: cost of illness
COR: consumption of resources
DM: myotonic dystrophy
DMD: duchenne muscular dystrophy
HRQOL: health-related quality of life
MDA: muscular dystrophy association
PedsQL™^©^: Pediatric Quality of Life Inventory™^©^
PRO: patient-reported outcomes
SD: standard deviation
SMA: spinal muscular atrophy
SMN1 gene: *survival motor neuron 1* gene
TREAT-NMD: translational research in Europe-assessment & treatment of neuromuscular diseases

## Footnotes

1 In Germany, the long-term care insurance is classified into three levels of care, thereby reflecting the grade of required personal care. Care allowance for at-home care delivered by relatives in 2013/2014 is calculated with €305, €525 and €700 per month for care level I, II and III, respectively. Alternatively, care rendered by professional care givers is covered by €665, €1,250, €1,550 and €1,918 per month for care level I, II, III and cases of hardship respectively [38].

## References

[1] Kolb SJ, Kissel JT (2015). Spinal Muscular Atrophy. Neurol Clin.

[2] Darras BT (2015). Spinal muscular atrophies. Pediatr Clin North Am.

[3] Lefebvre S, Burglen L, Reboullet S, Clermont O, Burlet P, Viollet L (1995). Identification and characterization of a spinal muscular atrophy-determining gene. Cell.

[4] D’Amico A, Mercuri E, Tiziano FD, Bertini E (2011). Spinal muscular atrophy. Orphanet J Rare Dis.

[5] Wang CH, Finkel RS, Bertini ES, Schroth M, Simonds A, Wong B (2007). Consensus statement for standard of care in spinal muscular atrophy. J Child Neurol.

[6] Schreiber-Katz O, Klug C, Thiele S, Schorling E, Zowe J, Reilich P (2014). Comparative cost of illness analysis and assessment of health care burden of Duchenne and Becker muscular dystrophies in Germany. Orphanet J Rare Dis.

[7] Varni JW, Seid M, Kurtin PS (2001). PedsQL 4.0: reliability and validity of the Pediatric Quality of Life Inventory version 4.0 generic core scales in healthy and patient populations. Med Care.

[8] Davis SE, Hynan LS, Limbers CA, Andersen CM, Greene MC, Varni JW (2010). The PedsQL in pediatric patients with Duchenne muscular dystrophy: feasibility, reliability, and validity of the Pediatric Quality of Life Inventory Neuromuscular Module and Generic Core Scales. J Clin Neuromuscul Dis.

[9] Iannaccone ST (2002). American Spinal Muscular Atrophy Randomized Trials G. Outcome measures for pediatric spinal muscular atrophy. Arch Neurocir.

[10] Iannaccone ST, Hynan LS (2003). American Spinal Muscular Atrophy Randomized Trials G. Reliability of 4 outcome measures in pediatric spinal muscular atrophy. Arch Neurocir.

[11] Iannaccone ST, Hynan LS, Morton A, Buchanan R, Limbers CA, Varni JW (2009). The PedsQL in pediatric patients with Spinal Muscular Atrophy: feasibility, reliability, and validity of the Pediatric Quality of Life Inventory Generic Core Scales and Neuromuscular Module. Neuromuscul Disord.

[12] Bladen CL, Thompson R, Jackson JM, Garland C, Wegel C, Ambrosini A et al. Mapping the differences in care for 5,000 Spinal Muscular Atrophy patients, a survey of 24 national registries in North America, Australasia and Europe. Journal of neurology. 2013. doi:10.1007/s00415-013-7154-1. 10.1007/s00415-013-7154-1 24162038

[13] Greiner W, Damm O, Schöffski O GvdSJ-M (2012). Die Berechnung von Kosten und Nutzen. Gesundheitsökonomische Evaluation.

[14] Bock JO, Brettschneider C, Seidl H, Bowles D, Holle R, Greiner W (2015). Calculation of standardised unit costs from a societal perspective for health economic evaluation. Gesundheitsw.

[15] Krauth C, Hessel F, Hansmeier T, Wasem J, Seitz R, Schweikert B (2005). Empirical standard costs for health economic evaluation in Germany -- a proposal by the working group methods in health economic evaluation. Gesundheitsw.

[16] Lauer-Fischer (2013). Die LAUER-Taxe 2013.

[17] Sozialleistungen (2012). Angaben zur Krankenversicherung.

[18] Norwood FL, Harling C, Chinnery PF, Eagle M, Bushby K, Straub V (2009). Prevalence of genetic muscle disease in Northern England: in-depth analysis of a muscle clinic population. Brain.

[19] The Federal Health Monitoring System: Health expenditures in Germany as share of GDP and in millions of Euro (absolute and per inhabitant). 2015. https://www.destatis.de/DE/Publikationen/Thematisch/Gesundheit/Gesundheitsausgaben/AusgabenGesundheitPDF_2120711.pdf?__blob=publicationFile. Accessed 30 Sep 2015.

[20] von Gontard A, Rudnik-Schoneborn S, Zerres K (2012). Stress and coping in parents of children and adolescents with spinal muscular atrophy. Klin Padiatr.

[21] Larkindale J, Yang W, Hogan PF, Simon CJ, Zhang Y, Jain A (2014). Cost of illness for neuromuscular diseases in the United States. Muscle Nerve.

[22] MDA. Cost of Amyotrophic Lateral Sclerosis, Muscular Dystrophy, and Spinal Muscular Atrophy in the United States - Final Report. Muscular Dystrophy Association, The Lewin Group Inc. 2012. http://www.mda.org/sites/default/files/Cost_Illness_Report.pdf. Accessed 03.08.2015.

[23] Reinhold T, Brüggenjürgen, Bernd, Schlander, Michael, Rosenfeld, Stephanie, Hessel, Franz, Willich, Stefan N. Economic analysis based on multinational studies: methods for adapting findings to national contexts. J Public Health. 2010;18(4):327–35. http://dx.doi.org/10.1007/s10389-010-0315-0.

[24] Zerres K, Davies KE (1999). 59th ENMC International Workshop: Spinal Muscular Atrophies: recent progress and revised diagnostic criteria 17–19 April 1998, Soestduinen, The Netherlands. Neuromuscul Disord.

[25] Cobben JM, Lemmink HH, Snoeck I, Barth PA, van der Lee JH, de Visser M (2008). Survival in SMA type I: a prospective analysis of 34 consecutive cases. Neuromuscul Disord.

[26] Landfeldt E, Lindgren P, Bell CF, Schmitt C, Guglieri M, Straub V (2014). The burden of Duchenne muscular dystrophy: An international, cross-sectional study. Neurol.

[27] Black N (2013). Patient reported outcome measures could help transform healthcare. BMJ.

[28] Guyatt GH, Ferrans CE, Halyard MY, Revicki DA, Symonds TL, Varricchio CG (2007). Exploration of the value of health-related quality-of-life information from clinical research and into clinical practice. Mayo Clin Proc.

[29] Varni JW, Burwinkle TM, Lane MM (2005). Health-related quality of life measurement in pediatric clinical practice: an appraisal and precept for future research and application. Health Qual Life Outcomes.

[30] Varni JW, Limbers CA, Burwinkle TM. Impaired health-related quality of life in children and adolescents with chronic conditions: a comparative analysis of 10 disease clusters and 33 disease categories/severities utilizing the PedsQL 4.0 Generic Core Scales. Health Qual Life Outcomes. 2007;5:43. doi:10.1186/1477-7525-5-43. 10.1186/1477-7525-5-43 PMC1964786 17634123

[31] Kaufmann P, McDermott MP, Darras BT, Finkel RS, Sproule DM, Kang PB (2012). Prospective cohort study of spinal muscular atrophy types 2 and 3. Neurol.

[32] de Oliveira CM, Araujo AP (2011). Self-reported quality of life has no correlation with functional status in children and adolescents with spinal muscular atrophy. Eur J Paediatr Neurol.

[33] Kocova H, Dvorackova O, Vondracek P, Haberlova J (2014). Health-related quality of life in children and adolescents with spinal muscular atrophy in the Czech Republic. Pediatr Neurol.

[34] Faravelli I, Nizzardo M, Comi GP, Corti S (2015). Spinal muscular atrophy--recent therapeutic advances for an old challenge. Nat Rev Neurol.

[35] Wertz MH, Sahin M. Developing therapies for spinal muscular atrophy. Ann N Y Acad Sci. 2015. doi:10.1111/nyas.12813. 10.1111/nyas.12813 PMC4713374 26173388

[36] Glossar P-Q. Pflegestufen. Bundesministerium für Gesundheit. 2015. http://www.bmg.bund.de/themen/pflege/pflegebeduerftigkeit/pflegestufen.html. Accessed 29 Jan 2016.

[37] Bevölkerung auf Grundlage des Zensus 2011. Statistisches Bundesamt. https://www.destatis.de/DE/ZahlenFakten/GesellschaftStaat/Bevoelkerung/Bevoelkerungsstand/Tabellen/Zensus_Geschlecht_Staatsangehoerigkeit.html. Accessed 18 Aug 2015.

[38] Pflegeleistungen ab 1. Januar 2015. Bundesministerium für Gesundheit. 2015. http://www.bmg.bund.de/fileadmin/dateien/Downloads/P/Pflegestaerkungsgesetze/Tabellen_Plegeleistungen_BRat_071114.pdf. Accessed 27. Jan. 2016.

